# Feasibility for Real-Time Monitoring of Bacterial Growth in Raw Milk Using a New Contactless Sensor

**DOI:** 10.1021/acs.analchem.5c03766

**Published:** 2025-10-30

**Authors:** Charles A. Haab, Jussiane S. Silva, Adriano M. Jaime, Vandré S. Pinto, Geovana M. Mello, Darliana M. Souza, Juliano S. Barin, Cristiano R. Menezes, Leandro Michels

**Affiliations:** † Departamento de Processamento de Energia Elétrica, Universidade Federal de Santa Maria, 97105-900 Santa Maria, Rio Grande do Sul, Brazil; ‡ Departamento de Química, 28118 Universidade Federal de Santa Maria, 97105-900 Santa Maria, Rio Grande do Sul, Brazil; § Departamento de Tecnologia e Ciência dos Alimentos, Universidade Federal de Santa Maria, 97105-900 Santa Maria, Rio Grande do Sul, Brazil

## Abstract

A novel method was developed for real-time quantification of the total bacterial count in raw milk using an electrical bacterial growth sensor based on a capacitively coupled contactless resonance frequency detector. The proposed method continuously monitors changes in the resonance frequency induced by bacterial metabolic activity, allowing for the construction of growth curves without requiring sample pretreatment or reagent addition. Growth curve analysis was performed using the Gompertz model, and the inflection point (β) was used to construct a predictive model for determining the total bacterial count. A total of 55 raw milk samples were used for the predictive model and application of the proposed method, which were compared to the standard plate count reference method. The predictive model demonstrated a good coefficient of determination (*R* ^2^ = 0.75). A comparative analysis between the proposed and reference methods showed no significant difference (*t*-test, 95% confidence level). The proposed method presented a limit of detection of 2.40 log CFU mL^–1^. The results also demonstrate that the proposed method presents a higher greenness score (score = 0.75) compared to the reference method (score = 0.39) and that the analysis time could be reduced from 48 to 8 h to classify the raw milk according to Normative Instruction No. 55/2020. These findings highlight the feasibility of the proposed method for rapid, green, and real-time monitoring of bacterial growth, allowing a promising alternative to microbiological quality control in the dairy industry.

## Graphical abstract

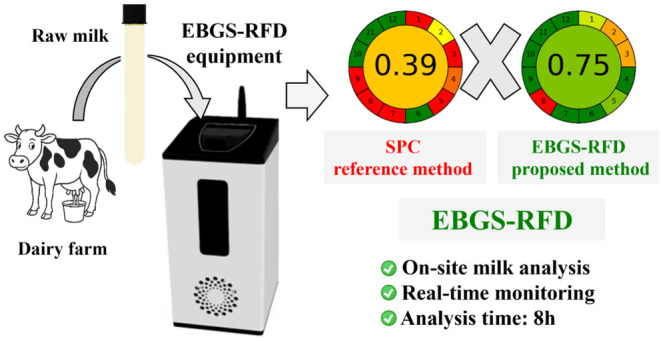

## Introduction

Milk is widely consumed in the world due to its high nutritional value and role as a significant source of essential macronutrients and micronutrients.^1,2^ Beyond its nutritional properties, milk plays a significant socioeconomic role, particularly in Brazil, where dairy production is essential for rural economies.^1,3^ Brazil ranks among the top ten milk-producing countries, with annual production exceeding 34 billion liters in 2022, accounting for approximately 4.5% of the global milk production of around 758 billion liters.^3^ Global milk production underscores the significant role of the dairy industry in ensuring food security and promoting economic development worldwide.^1,3^

However, the dairy sector faces persistent challenges related to milk quality, especially in microbiological control, which is critical for ensuring food safety and compliance with domestic and international market regulations.^1,3–6^ High bacterial contamination is associated with increased enzymatic activity and the degradation of key milk components, rendering it unsuitable for consumption and industrial processing.^7,8^ Although milk is sterile in the udder, microbial contamination is inevitable after milking due to the ubiquitous presence of microorganisms in the environment.^7,9,10^ Therefore, ensuring microbiologically safe raw milk without pasteurization or sterilization is practically not feasible.^9^ Thus, regulatory frameworks establish limits for total bacterial count (TBC) in milk, which indicate hygiene and refrigeration conditions during storage and transportation to the dairy industry.^8^

In Brazil, TBC is regulated by the Ministry of Agriculture, Livestock, and Supply (MAPA) and is expressed in colony-forming units per milliliter, CFU mL^–1^.^11^ According to the current regulatory framework (IN No. 55/2020), refrigerated raw milk at 7 °C must not exceed 300 000 CFU mL^–1^ before processing.^11^ Bacterial count regulations differ globally. In the United States, the threshold is 300 000 CFU mL^–1^ for mixed raw milk, with a stricter limit of 100 000 CFU mL^–1^ for grade A milk.^1^ The European Union mandates that raw milk for processing must not exceed 100 000 CFU mL^–1^,^12^ while England applies a more stringent limit of 20 000 CFU mL^–1^ based on the Standard Plate Count.^13^

Official methods for determining TBC in raw milk are based on standardized protocols, including the Standard Plate Count (SPC) method, as specified by ISO 4833-1.^14^ Regulatory agencies establish reference methods for microbial quantification, with Brazil adopting ISO 4833-1 and ISO 4833-2 for total bacterial count assessments in raw milk.^6,14,15^ Similarly, the European Commission uses ISO 4833-1 to determine TBC.^12,13^ Additionally, the U.S. Food and Drug Administration (FDA) and the European Commission employ ISO 4833-2 for microbial quantification in various food matrices, particularly milk and dairy products, reinforcing its international recognition.^16^ The SPC method involves serial dilution, inoculation onto culture media, incubation at 37 °C for 24 to 48 h, and manual colony counting.^6,14,15^ Despite its widespread use, the SPC method presents several limitations, including prolonged analysis time, the need for specialized laboratory infrastructure, and high reagent consumption.^6,15^ Moreover, its labor-intensive nature and high operational costs pose challenges, particularly for small-scale producers and industries that require rapid and cost-effective results.^17,18^ Therefore, there is a need to develop alternative methods that strike a balance between efficiency and simplicity.

Several alternative methods have been developed, including automated techniques such as flow cytometry (FCM),^19,20^ which provides rapid and accurate real-time bacterial analyses for high-throughput sample processing.^19,21–23^ The application of FCM to complex matrices such as raw milk is contingent upon prior sample preparation (e.g., clarification, centrifugation, or filtration), an essential step to mitigate interferences, and the employment of viability dyes, which enable the detection of viable but nonculturable cells.^24^ Although real-time polymerase chain reaction offers high analytical sensitivity and same-day results, its approach can detect DNA from nonviable cells, which necessitates careful validation in complex dairy matrices. Operationally, this technique also requires a greater investment in instrumentation and consumables when compared to the conventional SPC method.^25–27^ However, the high cost of the instrumentation and the inability to distinguish between viable and nonviable cells limit its adoption on a large scale, used mainly by small and medium-sized dairy farmers.^20,28,29^ In addition, spectroscopic approaches, such as near-infrared (NIR) spectroscopy and impedance spectroscopy, offer nondestructive and potentially more sustainable alternatives by eliminating the need for chemical reagents.^17,30^ However, their accuracy can be affected by the complexity of the milk matrix and the necessity for rigorous calibration, posing challenges to their implementation in the dairy industry.^30^ Microfluidic impedance sensors integrate fluid handling with interdigitated microelectrodes (IDAs) in tiny chambers, which increase the signal-to-noise ratio, minimize sample volumes, and speed up mass transport. The architecture of these sensors facilitates the incorporation of on-chip preconcentration methods, which enrich the sample by orders of magnitude, shorten detection times, and enable the use of multiple IDAs to further enhance sensitivity. Alternative preconcentration approaches, such as controlled microdroplet evaporation, can trigger bacterial osmoregulatory responses, enabling the discrimination between live and dead cells in minutes based on conductance without relying on growth kinetics. A disadvantage is the susceptibility to fouling and microchannel clogging in complex matrices (e.g., raw milk). In such matrices, proteins and lipids can adsorb onto the IDAs and channel walls, shifting the baseline impedance, degrading signal-to-noise, and necessitating surface passivation, rigorous cleaning routines, and more frequent calibration.^23^

Biosensor-based methods, including optical and DNA/RNA sensors, represent advancements in specificity and sensitivity for detecting target bacterial species.^31,32^ Nevertheless, these approaches face operational challenges, including high reagent and equipment costs and the requirement for sophisticated laboratory infrastructure.^32,33^ More recently, capacitive sensors have emerged as promising alternatives by addressing these limitations. These sensors detect changes in the dielectric properties of milk resulting from bacterial metabolism, allowing for real-time capacitance monitoring.^34–36^ Compact, portable, and user-friendly capacitive sensors eliminate the need for reagents or sample preparation, mitigating issues related to the degradation of biological components.^34,35^

Advances in impedance microbiology have further accelerated microbial detection by identifying biochemical changes in the medium, such as variations in milk dielectric properties during bacterial proliferation.^34,35,37^ Contactless methods, such as the electrical bacterial growth sensor (EBGS), have been introduced to minimize contamination risks associated with direct electrode contact with the sample.^34,35^ Although effective, conventional EBGS devices require a fixed frequency and depend on high-precision circuit components such as signal generators, transimpedance amplifiers, and peak detectors to resolve small variations in the signal.^35^ In contrast, an Electrical Bacterial Growth Sensor based on a capacitively coupled, contactless resonance frequency detector (EBGS-RFD) was developed.^38,39^ The central innovation of this device lies in inherently maximizing signal gain and simplifying the electronics, thereby eliminating the need for high-bandwidth amplifiers or traditional peak detectors. In this design, capacitively coupled external electrodes around a glass tube containing the milk sample monitor shifts in resonance frequency caused by medium composition changes, enabling real-time detection of bacterial growth.^34,38,39^

This study introduces an innovative approach for the real-time quantification of TBC in raw milk using a novel EBGS-RFD. The novelty of this proposed method is based on the ability to monitor bacterial growth without direct contact with the sample and without the addition of chemical reagents, addressing the main limitations of the conventional method. The main objective was to develop a predictive model based on the analysis of bacterial growth curves using the inflection point (β) as a key parameter. To demonstrate its viability, the performance of the EBGS-RFD was calibrated by correlating the β with results obtained using the reference method (SPC method, ISO 4833-2). The accuracy of the EBGS-RFD proposed method was determined by comparing its results to those of the SPC reference method. Finally, this work aims to present a rapid, automated tool for microbiological quality control, contributing to the dairy industry and aligning with the principles of Green Analytical Chemistry, enabling faster, more sustainable, and reagent-free analyses.

## Methods

### Samples and Reagents

In this study, 55 raw milk samples were collected over one year from the dairy production sector of the Federal University of Santa Maria (UFSM). This extended collection period was designed to incorporate the natural seasonal variability in both the milk composition and its native microbiota, ensuring the development of a robust predictive model. Samples were collected in sterile containers (2 L), transported under controlled temperature conditions, and stored at 5 °C until analysis, in accordance with Normative Instruction No. 55 (IN 55/2020)^11^ issued by the MAPA of Brazil. The chemical composition of the raw milk samples was determined following the official methods established by the MAPA, IN 77/2018.^6^ The official methods are presented in the Supporting Information (S1. Official Methods for Chemical Composition Analysis of Raw Milk). From the analyses, the results obtained were 3.20 ± 0.10% for protein, 3.52 ± 0.15% for fat, 4.50 ± 0.12% for carbohydrates, and 88.1 ± 0.42% for water (determined from the analysis of total solids).

The analytical-grade reagents included 0.1% (v v^–1^) buffered peptone water (Dinâmica Qumica Contemporânea Ltd., Brazil) for sample dilution and Plate Count Agar (PCA, HiMedia, India) as the solid culture medium. The PCA medium was prepared according to ISO 4833-2:2013.

### Standard Plate Count (SPC)

The SPC method was used as the reference for TBC, following the guidelines outlined in ISO 4833-2:2013 ^6,15^ and IN 30.^40^ The results obtained were used as a reference for evaluating the EBGS-RFD device (BioAiLab, Auftek, Brazil).^34^

Raw milk samples were homogenized before dilution. A 1 mL aliquot was diluted in 9 mL of 0.1% (v v^–1^) buffered peptone water. Serial 10-fold dilutions were prepared to ensure colony counts within the 25 to 250 range, as recommended by the Food and Drug Administration (FDA, 2001). For each dilution, 100 μL was plated onto preprepared PCA plates using the surface spread plate technique. After complete absorption of the inoculum, the plates were inverted and incubated at 36 ± 1 °C for 48 h in an oven (SSBI 21L, SolidSteel, Brazil). Following incubation, visible colonies were counted using a colony counter (LGI-CC-30, LaborGlas, Brazil), estimating the viable bacterial population in the original raw milk sample. The results were expressed in colony-forming units per milliliter (CFU mL^–1^).^41^

All analyses were conducted in triplicate. All materials were sterilized in a vertical autoclave (CS 75, Prismatec, Brazil) at 121 °C and 1.2 atm for 20 min before use to eliminate potential microbial contaminants.

### Electrical Bacterial Growth Sensor Based on a Capacitively Coupled Contactless Resonance Frequency Detector (EBGS-RFD)

The analysis of raw milk samples was performed using the EBGS-RFD device (BioAiLab, Auftek, Brazil), which features a resonance frequency-based sensor capable of generating bacterial growth curves.^34^ The EBGS-RFD system incorporates a contactless impedance microbiology approach utilizing a measurement chamber with capacitive coupling. The chamber comprises a capacitive coupling system formed by two axially aligned conductive rings separated by a 5 mm gap. These stainless steel rings, measuring 16 mm × 16 mm × 1 mm (Figure S1, Supporting Information), are coupled to the test tube containing the raw milk sample. The device was designed for standard 10 mL glass tubes commonly utilized in laboratory settings.^34,38,39^

To ensure technical clarity and reproducibility, the performance parameters of the EBGS-RFD sensor, along with its detailed design, are presented in a previous work.^34^ Sensor sensitivity was maximized through simulations using MAXWELL software (2022 version) to optimize electrode dimensions. Operation at the resonant frequency of the system results in a high signal-to-noise ratio, as evidenced by a relative standard deviation below 0.01% during measurements. Additionally, thermal drift is negligible because both the sensor and the sample are housed within a temperature-controlled chamber.^34^ The signal processing flowchart and the detailed electrical model are presented in a previous work,^34^ since the main objective of this work is to present the development of a method for the real-time quantification of TBC in raw milk using a novel EBGS-RFD.

The EBGS-RFD method for determining TBC in raw milk was evaluated based on the analysis of bacterial growth curves. These curves were analyzed using the Gompertz mathematical model, with key parameters extracted as outlined in the workflow presented in Figure 1. Data acquisition was performed continuously since the introduction of the tube into the measurement chamber, with a sampling rate of one reading per minute. The bacterial growth curves exhibited three distinct phases: the lag phase, the exponential phase, and the stationary phase, thereby allowing for real-time identification of microbiological behavior. The experimental procedure consisted of adding 10 mL of previously homogenized raw milk to a glass tube containing a magnetic stir bar without adding any reagent. The glass tube was sealed with an autoclavable Bakelite cap, ensuring sterile conditions throughout the experiment. All materials utilized were previously sterilized, as described in Section Standard Plate Count (SPC) section. Sample incubation was performed directly within the EBGS-RFD device at a controlled temperature of 36 ± 0.3 °C, in compliance with the specifications of Normative Instruction No. 30/2018.^40^

**Figure 1:**
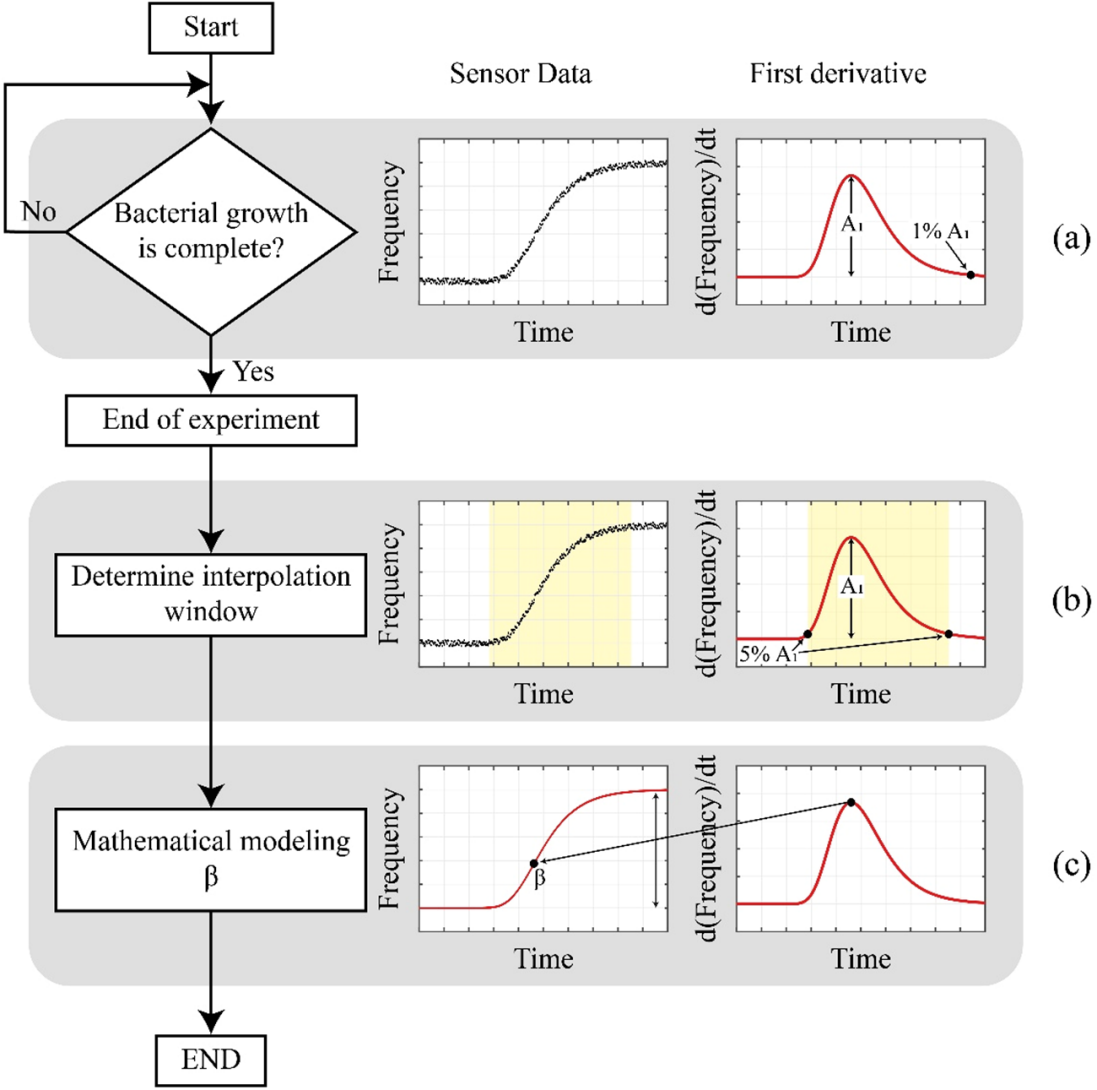
Workflow of the algorithm applied in the EBGS-RFD method for the determination of TBC in raw milk: (a) bacterial growth monitoring, (b) identification of the interpolation window, and (c) mathematical modeling of β.

The algorithm applied in the EBGS-RFD method for determining TBC in raw milk was divided into three principal stages: bacterial growth monitoring (Figure 1a), identification of the interpolation window (Figure 1b), and mathematical modeling of parameter β (Figure 1c). In the first step (Figure 1a), corresponding to bacterial growth monitoring, the sensor continuously collected one data point per minute, thereby capturing the resonance frequency changes associated with bacterial proliferation in the raw milk sample. The first derivative of the frequency data, d­(frequency)/dt, was calculated to determine the frequency change rate over time. This procedure enabled the identification of the moment when the rate decreased significantly, which indicated the termination of the logarithmic phase of the bacterial growth curve. This analysis was performed in real time, allowing for identification of the maximum growth rate (A1). The end of the logarithmic phase was defined as the moment when the growth rate reached 1% of A1.

In the subsequent stage, an interpolation window was defined to capture the relevant data from the bacterial growth curve. This window was delimited by the time interval corresponding to 5% of A1 on both sides of the growth curve (yellow region in Figure 1b).

The frequency data within the interpolation window were fitted to the Gompertz curve, which is widely used to describe bacterial growth under controlled conditions (Tribst et al., 2019; J. Wang and Guo, 2024). The curve is mathematically represented by

1 $$f\left(t\right)=Ae^{Be^{C\left(t+D\right)}}+E$$

where *A*, *B*, *C*, *D*, and *E* are parameters estimated through nonlinear regression. Following the model fitting, the inflection point (β) was calculated based on the condition of maximum growth rate. The value of β is given by

2 $$\beta =\frac{1}{C}\ln\left(-\frac{1}{B}\right)-D$$

The β parameter from the bacterial growth curve was employed to construct a predictive model for TBC, expressed in log CFU mL^–1^, as determined by the standard SPC method. The model calibration was based on 55 raw milk samples, which were analyzed in parallel by using the proposed EBGS-RFD and the SPC reference methods. The performance of the predictive model was evaluated using statistical metrics, including the coefficient of determination (*R* ^2^) and the root-mean-square error (RMSE).

An independent analysis was conducted to further assess the accuracy of the EBGS-RFD method. For this purpose, eight raw milk samples were analyzed using both the EBGS-RFD and the SPC reference method (ISO 4833-2:2013a).^15^ The total bacterial count obtained by each method was compared using a paired Student *t*-test with a 95% confidence level and triplicate measurements for each sample.

## Results and Discussion

### Evaluation of the EBGS-RFD Method for TBC Determination in Raw Milk

The analysis of TBC by EBGS-RFD was performed based on the conditions recommended by the standard method outlined in the regulations of the MAPA.^6^ A 10 mL aliquot of raw milk, without dilution, was placed directly into standard glass test tubes, which were sealed and inserted into the EBGS-RFD device. The raw milk samples were incubated at 36 °C in the device without adding culture media or supplemental reagents. Once the incubation temperature stabilized, it was maintained throughout the experiment. The resonance frequency was measured at 1 min intervals, enabling the construction of bacterial growth curves over time for the analyzed raw milk samples. These curves were analyzed using the Gompertz mathematical model, and their key parameters were estimated following the workflow presented in Figure 1. The process for estimating the β parameter is shown in Figure 2; four raw milk samples from the calibration set were selected, each with a different contamination level: 6.84 (purple), 3.93 (yellow), 1.69 (red), and 1.38 (blue) log CFU mL^–1^.

**Figure 2:**
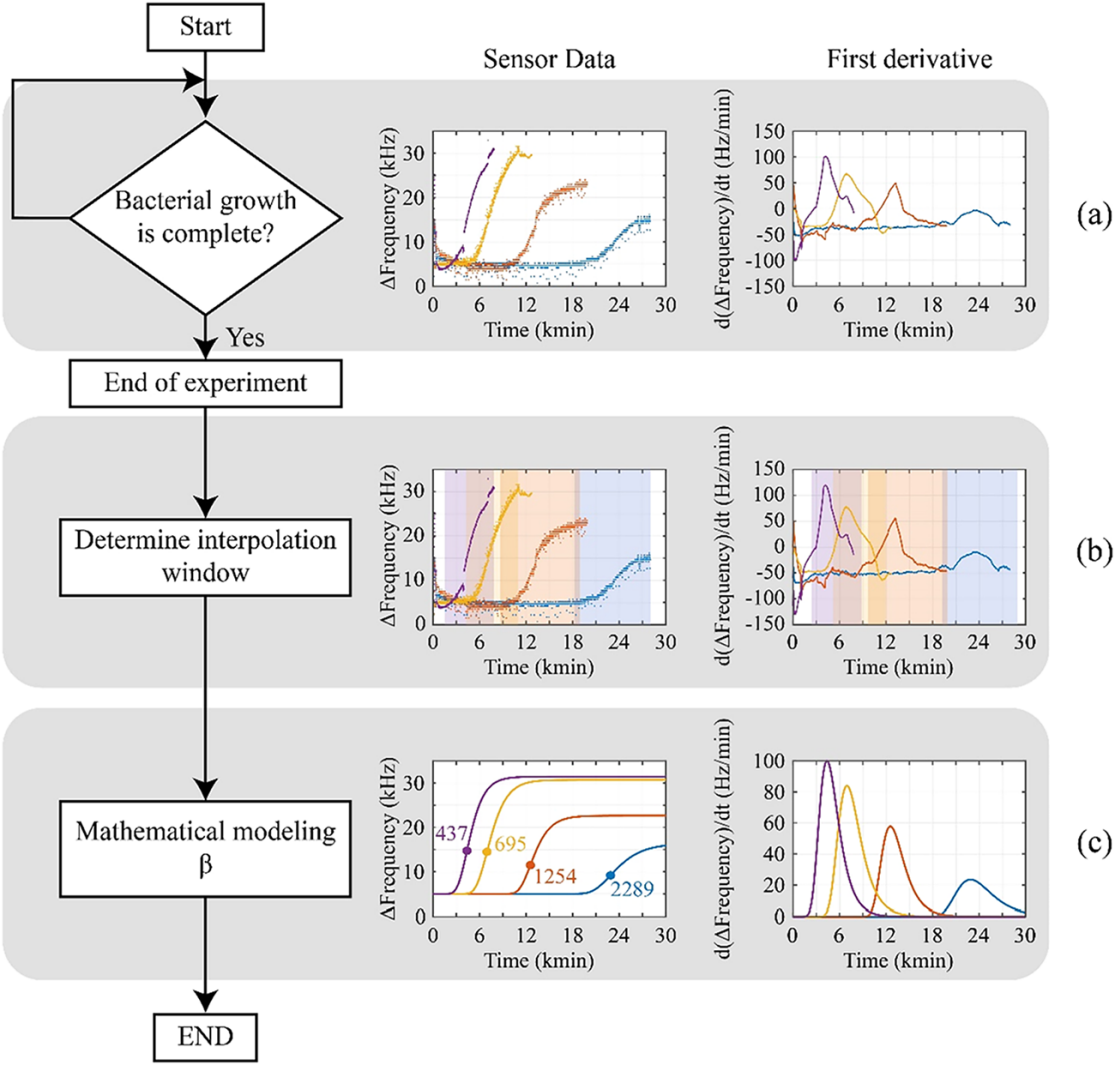
Workflow for estimating the β parameter from bacterial growth curves using the EBGS-RFD method: (a) real-time monitoring of bacterial growth through frequency variation (left) and its first derivative (right); (b) identification of the interpolation window based on the derivative signal; and (c) the fitting of the Gompertz model within the selected window to determine the inflection point β. Data correspond to raw milk samples with contamination levels of 6.84 (purple), 3.93 (yellow), 1.69 (red), and 1.38 (blue) log CFU mL^–1^.

To confirm that the signals detected by the EBGS-RFD originate from bacterial metabolic activity and not from nonbiological particulate interferences, such as fat globules or protein aggregates, a control experiment was conducted. As detailed in a previous work,^34^ a sterile milk sample (negative control) was analyzed in parallel with the untreated samples under identical conditions. The results demonstrated that while the contaminated samples exhibited characteristic sigmoidal growth curves, the sterile control sample presented a stable frequency baseline with no significant variations over the incubation period. This finding confirms that the measured dynamic signal is specific to microbial growth and the consequent metabolic alteration of the medium, thereby ruling out false positives originating from the milk matrix.

As shown in Figure 2a, bacterial growth profiles varied according to the contamination levels. Higher contamination led to a faster transition into the logarithmic phase, whereas lower contamination resulted in a prolonged lag phase before exponential growth began. This pattern reflects the microbial adaptation process, where samples with a more significant initial bacterial load exhibit a more rapid cell multiplication with consequent fast change in the composition of the medium. In contrast, bacteria in low-contamination samples require more time to provide changes in the composition of the medium for sensor detection.

The first derivative analysis of the signal allowed for the identification of interpolation windows for each growth curve (Figure 2b). This step highlighted the point where the rate of frequency variation decreased significantly, indicating the transition between growth phases. Once the interpolation window was defined, the data were fitted to the Gompertz curve, enabling the determination of the inflection point (β). Following the Gompertz model adjustment, parameters A–E were estimated for each bacterial growth curve (Figure 2c). The β parameter was calculated by using eq 2 to correlate the EBGS-RFD method with bacterial counts determined by the SPC reference method. The estimated parameters and their 95% confidence intervals for the four raw milk samples with varying contamination levels are listed in Table 1.

**Table 1:** Gompertz Model Parameters for Bacterial Growth Curves in the Raw Milk Samples

sample	1	2	3	4
TBC, log (UFC mL^–1^)	6.84	3.93	1.69	1.38
A	33.19 ± 1.48	32.28 ± 0.30	22.15 ± 0.37	14.28 ± 0.53
B	–0.1361 ± 0.0324	–0.3037 ± 0.0125	–0.1083 ± 0.0160	–0.2238 ± 0.0345
C	–0.0081 ± 0.0008	–0.0070 ± 0.0002	–0.0070 ± 0.0004	–0.0044 ± 0.0003
D	–683.63 ± 0.00	–865.59 ± 0.00	–1571.95 ± 0.00	–2627.08 ± 0.00
E	1653.95 ± 0.42	785.13 ± 0.12	1357.77 ± 0.15	1492.34 ± 0.06
β, min	437.25 ± 4.09	695.03 ± 1.41	1254.04 ± 1.41	2288.64 ± 6.18

The results in Table 1 demonstrate that the EBGS-RFD method effectively differentiated among various levels of bacterial contamination, as indicated by the analysis of growth curves and the β parameter. An inverse relationship was observed between β and the initial microbial load, confirming that samples with lower contamination require more time to reach the inflection point of the growth curve. In this way, the bacteria growth can only be detected if a change in the medium is enough to provide a difference in the sensor signal, which is related to the metabolism of a certain amount of bacteria cells. For example, primary metabolites such as lactic acid and certain amino acids can be released in the medium due to the growth of bacteria, with consequent changes in the physical properties of the solution inside the tube, allowing detection. These findings highlight the potential of the EBGS-RFD method for rapid and reagent-free assessment of microbial growth, offering a quantitative approach to evaluating bacterial growth dynamics in raw milk.

### Predictive Model Evaluation for the EBGS-RFD Method

The predictive model of the EBGS-RFD method was developed using 55 raw milk samples collected on different days over 12 months. To impartially evaluate the model performance, the set of 55 samples was divided into a training subset (*n* = 39, ∼70%) and a test subset (*n* = 16, ∼30%). The division was performed using a computational random sampling algorithm with a fixed random seed of 42 to ensure the reproducibility of the analysis and to avoid the risk of overfitting from data selection. This data set was randomly divided into two subsets: a training set of 39 samples used for model fitting and a test set of 16 samples used to evaluate the performance of the model. The samples were analyzed in parallel using an EBGS-RFD device and the SPC method. As shown in Figure 3, a correlation was established for each milk between the inflection point (β) of the bacterial growth curve obtained by the EBGS-RFD and the TBC measured by the SPC method.

**Figure 3:**
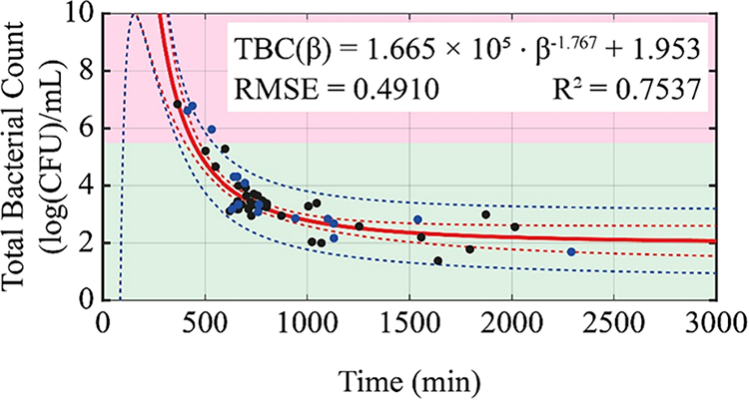
Predictive model relating the inflection point (β) obtained from the EBGS-RFD method to the TBC as determined by the SPC method for 55 raw milk samples. The red line represents the fitted model, and the dashed blue line indicates the 95% confidence interval. Black points correspond to the 39 samples used for model fitting (training set), and blue points represent the 16 samples used for model test (test set), following a 70-to-30% split.

The nonlinear regression model shown in Figure 3 is described by the equation CFU­(β) = 1.665 × 10^5^ × β^–1.767^ + 1.953, with an *R* ^2^ of 0.7537, indicating that approximately 75% of the variability in TBC values is explained by the model. This result reflects a good fit, especially considering the inherent biological variability.^8,42^

The statistical analysis demonstrated that the EBGS-RFD model consistently estimates the TBC, showing good accuracy and precision. The model presented a mean absolute error of 0.40 log CFU mL^–1^ (±0.32) and a mean relative error of 12.53% (±11.60%). The root-mean-square error (RMSE) was 0.51 log CFU mL^–1^, indicating a moderate dispersion between the values estimated by the model and those obtained by the SPC method. A comprehensive evaluation of the performance of the model and the agreement between the methods is presented in Figure 4. The Bland–Altman plot (Figure 4a) shows that most differences between the EBGS-RFD and SPC methods fall within the 95% limits of agreement, indicating no significant systematic bias. Furthermore, analysis of the residuals confirmed the validity of the model assumptions. The plot of residuals versus estimated values (Figure 4c) and versus the time parameter β (Figure 4b) shows a random distribution around zero, indicating no evidence of heteroscedasticity or systematic trends. The normal *Q*–*Q* plot (Figure 4d) suggests that the residuals are normally distributed. The variability at lower concentrations may be associated with both model limitations and uncertainties inherent to the reference method, such as errors in serial dilutions and manual colony counting.

**Figure 4:**
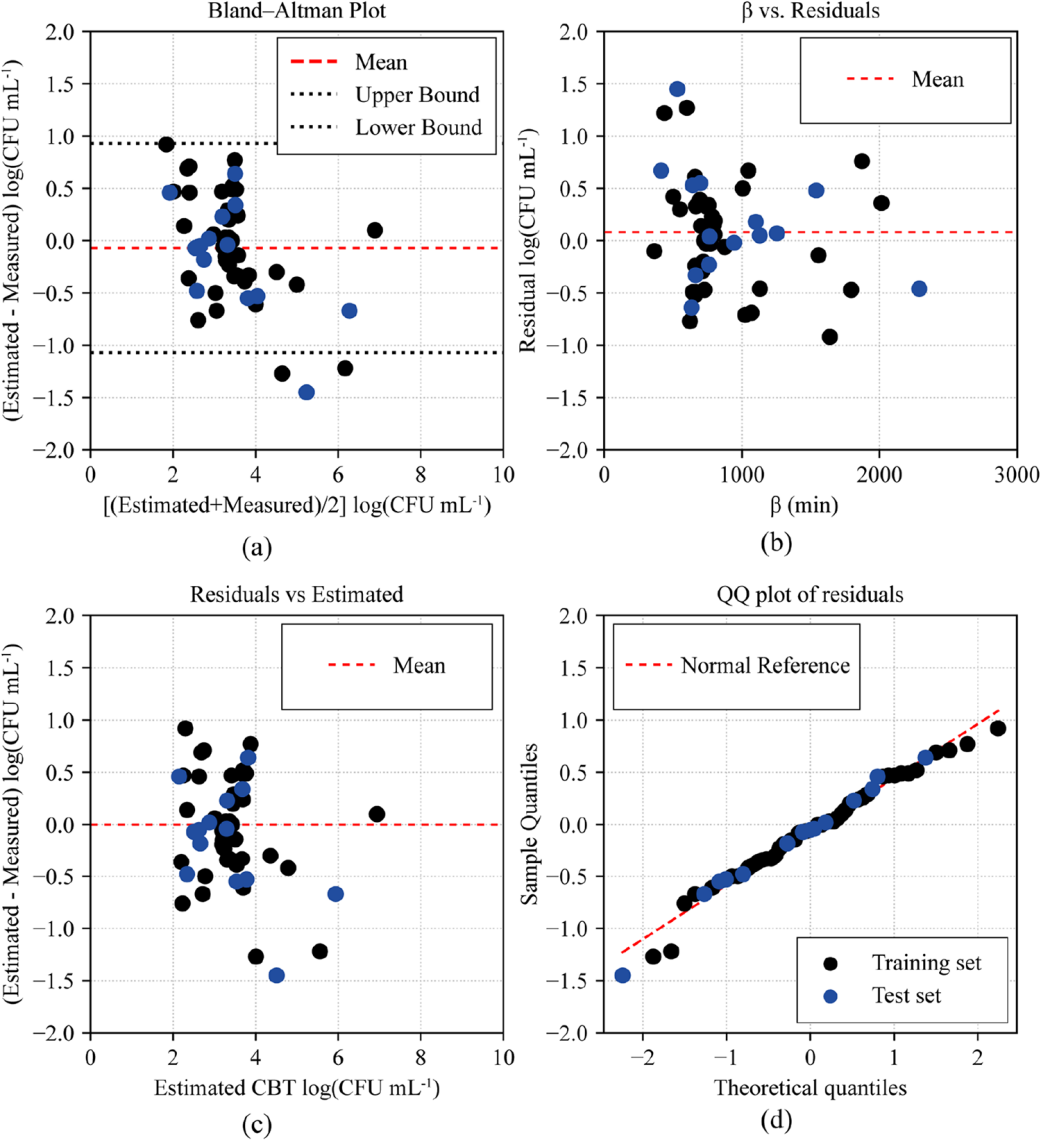
(a) Bland–Altman plot of the difference (estimated—measured, log­(CFU mL^–1^)) versus the average of the two methods ([(estimated + measured)/2], log­(CFU mL^–1^)); the red dashed line is the mean bias and black dotted lines are the 95% limits of agreement. (b) β vs residuals to assess error dependence on the time parameter β (min); the red dashed line marks the mean residual. (c) Residuals vs estimated TBC (log­(CFU mL^–1^)) to check functional form and constant variance; the red dashed line marks zero residual. (d) Normal *Q*–*Q* plot of residuals with group-specific normal reference lines. Points show all 55 samples (training set: black circles; test set: blue circles).

Furthermore, for β values exceeding 1500 min, the model indicates a reduction in sensitivity, particularly in samples with slow bacterial growth or low initial contamination levels, suggesting a detection limit under these conditions. To further define the analytical performance, the limit of detection (LOD) was estimated based on the parameters of the model curve, following the method described by Sazaklioglu et al.^43^ and recommended by IUPAC.^44^ First, the TBC­(β) curve was inverted to form β­(TBC). This inverse function was applied to the reference concentrations to obtain the predicted times. From these data, the standard deviation of the intercepts (*S*_β_) and the slope of the calibration curve (*m*) were estimated over a range of 24 h. Using the 3.*3 × S*_β_/*m* criterion, the LOD was 2.4 log CFU mL^–1^, approximately 250 CFU mL^–1^. The EBGS-RFD monitors chemical changes in the sample that reflect the metabolic dynamics of its microbiota.^8^ Milk is a complex microbial ecosystem where various bacterial species and strains interact cooperatively and competitively.^8,42^ Unlike conventional plate counting methods, where a given CFU value corresponds directly to the bacterial load, the same CFU measurement in EBGS-RFD may result in different growth profiles due to inherent metabolic variations among microbial populations.^8,10,42^ These metabolic fluctuations influence the medium composition, increasing the variability in determining growth curve β. This effect is particularly pronounced at high β values, where microbial activity is lower, leading to a more significant uncertainty in the TBC estimation.

One of the most relevant aspects of the EBGS-RFD method is the time required to determine the β. While the SPC reference method requires up to 48 h of incubation to develop visible colonies, the EBGS-RFD method can provide an initial evaluation in 480 min (8 h), enabling a preliminary classification of raw milk based on TBC levels. Additionally, the system facilitates real-time monitoring, providing continuous data acquisition throughout the incubation period. This enables the early identification of whether TBC values fall below the regulatory threshold, thereby supporting informed decision-making. This faster detection capability is particularly relevant within the raw milk supply chain, where delays may compromise product quality and food safety.

### Potential Applications of the EBGS-RFD Method in the Dairy Production Process

The EBGS-RFD can be used on dairy farms and at industrial reception points to rapidly assess TBC in raw milk, offering a practical and efficient alternative to conventional methods. To evaluate the application of the EBGS-RFD method, raw milk samples were collected from the dairy production sector of the UFSM. A total of eight raw milk samples were analyzed using EBGS-RFD, and the results were compared with those obtained by the SPC reference method to assess the accuracy of the proposed method. The TBC values obtained by both methods are presented in Table 2.

**Table 2:** Results of TBC in Raw Milk Using the EBGS-RFD Method and the SPC Reference Method^a^

sample	EBGS-RFD	SPC	*p* (Welch)
A	2.15 ± 0.20	1.69 ± 0.21	0.0516
B	2.63 ± 0.15	2.27 ± 0.18	0.0582
C	2.51 ± 0.06	2.58 ± 0.10	0.3691
D	2.63 ± 0.12	2.68 ± 0.05	0.5582
E	2.34 ± 0.09	2.82 ± 0.28	0.0856
F	2.66 ± 0.08	2.84 ± 0.18	0.2194
G	2.88 ± 0.11	2.86 ± 0.08	0.8127
H	3.31 ± 0.13	3.08 ± 0.18	0.1543
I	3.82 ± 0.08	3.18 ± 0.42	0.1138
J	3.30 ± 0.04	3.34 ± 0.07	0.4501
K	3.68 ± 0.11	3.34 ± 0.18	0.0610
L	3.54 ± 0.16	4.09 ± 0.17	0.0152
M	3.93 ± 0.35	4.31 ± 0.15	0.1920
N	3.78 ± 0.17	4.31 ± 0.09	0.0170
O	4.51 ± 0.15	5.96 ± 0.14	0.0003
P	5.94 ± 0.29	6.61 ± 0.06	0.0519

a Results are expressed as the mean ± standard deviation in log CFU mL^–1^ (*n* = 3). The *p*-value for each pair was determined using a two-tailed Welch *t*-test.

A comparative analysis between the EBGS-RFD and SPC methods for TBC in 16 raw milk samples indicated no significant systematic bias. The paired *t*-test indicated a mean difference between the methods of −0.147 log CFU mL^–1^ (95% confidence level [−0.426, 0.133]; *t*(15) = −1.120; *p* = 0.280). In individual per-sample comparisons, samples L, N, and O initially showed *p*-values < 0.05. After applying the Benjamini–Hochberg correction to control the false discovery rate for multiple comparisons, only sample O (*p* = 2.63 × 10^–4^) remained statistically significant. For a more rigorous assessment, a Two-One-Sided Tests (TOST) procedure for equivalence was performed, with an interval of ±0.45 log CFU mL^–1^, based on the reproducibility limit of the reference method ISO 4833-1. An α = 0.05 was adopted for each one-sided test (lower limit and upper limit), and a 90% confidence level was used for the mean difference (1–2α = 90%), which was [−0.377, 0.083] log CFU mL^–1^. The results for both the lower bound test (*t*(15) = −4.552, *p* = 0.0002) and the upper bound test (*t*(15) = 2.312, *p* = 0.0177) were significant. As the 90% confidence level was contained within these predefined limits, the EBGS-RFD and SPC methods are considered statistically equivalent under the conditions of this study.

The TBC results presented in Table 2 indicate that most of the analyzed samples fall within the acceptable limits for both Brazilian^11^ and United States regulations,^45^ with a maximum allowed TBC of 300 000 CFU mL^–1^ (5.48 log CFU mL^–1^). However, samples P (5.94 log CFU mL^–1^ by EBGS-RFD; 6.61 log CFU mL^–1^ by SPC) and O (4.51 log CFU mL^–1^ by EBGS-RFD; 5.96 log CFU mL^–1^ by SPC) present TBC values exceeding 300 000 CFU mL^–1^, indicating noncompliance with the regulatory limits established in Brazil and the United States. Furthermore, these samples also exceeded the maximum allowed TBC values defined by the European Union, 100 000 CFU mL^–1^.^12^ In addition, samples A, B, F, G, and H presented TBC values below 4.30 log CFU mL^–1^, meeting the strict limit established by the Food Standards Agency in England.^13^ This confirms the performance of the EBGS-RFD method in operating within the regulatory range for raw milk classification based on international quality standards.

The on-farm application of EBGS-RFD enables early screening of raw milk quality before collection. This real-time assessment allows for rapid identification of hygiene failures, bacterial contamination, and potential mastitis.^34,46,47^ The system delivers immediate results that support corrective actions by eliminating the need for laboratory infrastructure.^34^ These include detecting contaminated milking equipment, adjusting cleaning protocols, or isolating milk from infected animals.^46–48^ Such proactive monitoring reduces the risk of bulk tank contamination, enhances herd health management, and improves the overall microbiological quality before transport to industry.

In the dairy industry, the EBGS-RFD can be integrated into milk reception stations for immediate batch classification. Real-time analysis without needing laboratory infrastructure minimizes delays and prevents noncompliant milk from entering the processing line.^34^ This rapid decision-making process aligns with current food safety standards, quality assurance, and traceability in dairy production.^46,49^

### Greenness Score Evaluation of the EBGS-RFD and SPC Methods

The environmental impact and sustainability of the EBGS-RFD and SPC methods were evaluated using the Analytical GREEnness (AGREE) metric, which assesses compliance with the 12 principles of green analytical chemistry (GAC). The AGREE tool provides a pictogram-based visual representation of the overall score, ranging from 0 to 1, where higher values indicate greater adherence to green chemistry principles. The input data regarding the sample preparation methods used in both approaches were entered into the AGREE software and are presented in Table S1, Supporting Information. These data highlight the differences between the two methods (Table 3), emphasizing the simplified procedure and lower environmental impact of the EBGS-RFD method compared to those of the conventional approach (SPC method).

**Table 3:** Comparison of Figures of Merit between the SPC Reference Method and the EBGS-RFD Proposed Method for Quantifying TBC in Raw Milk

parameters	SPC	EBGS-RFD
sample throughput	24–48 h	8 h
apparatus	autoclave	pippete
	incubator	glass tube
	Petri dishes	EBGS-RFD system
	pippete	
	colony-counting equipment	
	tubes	
	drigalski spatula	
reagents	plate count agar (PCA), composition: pancreatic digest of casein, yeast extract, dextrose, sgar, deionized Water.	
sample pretreatment	agar preparation	analysis
	dilution	
	inoculate	
	incubate	
	count	

As shown in Table 3, the EBGS-RFD method eliminates the need for laboratory infrastructure, as it requires only a pipet, a glass tube, and the EBGS-RFD system, while SPC demands multiple pieces of equipment, including an autoclave, incubator, Petri dishes, colony-counting equipment, and reagents. The reduced apparatus requirements of EBGS-RFD contribute to lower energy consumption and fewer disposable materials, reinforcing its environmental benefits. As shown in Figure 5, the EBGS-RFD method achieved a score of 0.75 (Figure 5a), whereas the SPC method scored 0.39 (Figure 5b), confirming the superior performance of the proposed method. The EBGS-RFD method reduces sample treatment, adhering to Principle 1, as it enables direct analysis of raw milk without requiring pretreatment or reagent addition. In contrast, the SPC method demands extensive sample preparation, including dilution, inoculation, and incubation steps, increasing the process complexity and resource consumption. This difference is also presented in Table 3, where the SPC method requires Plate Count Agar (PCA) containing pancreatic digest of casein, yeast extract, dextrose, and agar, while the EBGS-RFD method operates without any reagents, minimizing chemical waste and reducing environmental impact.

**Figure 5:**
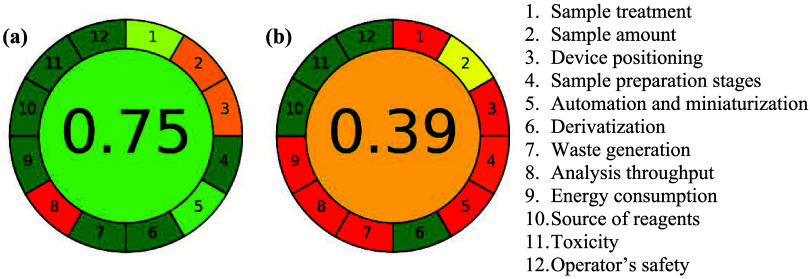
Results of AGREE analysis for the EBGS-RFD proposed method (a) and the SPC reference method (b).

The EBGS-RFD is an at-line method, aligning with Principle 3, allowing real-time monitoring of bacterial growth at or near the milk production site. On the other hand, the SPC is an offline method requiring a laboratory infrastructure; the EBGS-RFD can be operated outside of traditional lab settings, eliminating the need for controlled environments or specialized equipment. Furthermore, the straightforward handling process of the EBGS-RFD method allows nonspecialist personnel to collect and analyze samples with simple instructions, making it a practical and accessible tool for on-site microbiological monitoring in dairy farms and processing facilities. Table 3 highlights that the SPC method involves multiple handling steps, such as agar preparation, sample dilution, and colony counting, which introduce potential sources of error and variability. In contrast, the EBGS-RFD method simplifies the procedure by reducing the number of steps to direct sample analysis.

Another advantage of the EBGS-RFD method is its high level of process integration, which supports Principle 4. On the other hand, the SPC method requires at least eight distinct analytical steps. In contrast, the EBGS-RFD method enables analysis with only three main steps: sample homogenization, transfer to a glass tube, introduction into the equipment for incubation, and real-time acquisition of the bacterial growth curve. As outlined in Table 3, SPC requires labor-intensive processes, increasing operator workload, and extending the analysis time to 24–48 h. The EBGS-RFD method, on the other hand, provides results within 8 h, significantly reducing the time required for microbial assessment and enabling faster decision-making in quality control. This simplified procedure eliminates the need for extensive sample preparation, reducing both operator workload and potential sources of error. Additionally, the lower number of procedural steps directly minimizes energy consumption and reagents, reinforcing the sustainability and efficiency of the EBGS-RFD method compared with the SPC method.

Overall, Table 3 presents information that the EBGS-RFD method is operationally efficient and environmentally advantageous due to its reduced energy and resource consumption. The integration of real-time analysis, minimal reagent use, and reduced sample handling steps directly aligns with the principles of Green Analytical Chemistry, further supporting its potential as a sustainable alternative to conventional methods. The AGREE analysis confirms that the EBGS-RFD method offers significant environmental and operational advantages over the SPC method. By reducing sample treatment, integrating analytical processes, and utilizing automation, the procedure is simplified, energy consumption is decreased, and reagent use is eliminated, making it a greener, more efficient, and sustainable alternative for quantifying CBT in raw milk.

## Conclusions

This study developed a method for determining TBC in raw milk using EBGS-RFD. The sensor demonstrated a consistent analytical performance under controlled incubation conditions (36 ± 0.3 °C). The Gompertz model enabled the estimation of the inflection point (β), which is defined as the moment of maximum growth rate and obtained through nonlinear fitting of the frequency curve. Compared to the SPC reference method, the EBGS-RFD reduced analysis time from 48 to approximately 8 h, supporting more rapid decision-making in raw milk quality control. In addition to analytical performance, the EBGS-RFD method showed advantages in operational simplicity and environmental sustainability. Minimum sample preparation, reduced need for technical training, and elimination of chemical reagents contributed to lower variability and reduced environmental impact. Overall, the EBGS-RFD represents a practical alternative for routine microbial analysis in dairy sites, aligning with international regulations and principles of green analytical chemistry.

## Supplementary Material



## Notes

Official methods for chemical composition analysis of raw milk (Figure S1); electrical bacterial growth sensor based on a capacitively coupled contactless resonance frequency detector (EBGS-RFD, BioAiLab, Auftek) used for real-time quantification of TBC in raw milk (Table S1); information entered into the AGREE software as input data about the sample preparation methods used (PDF)
