# Supplementary material for: Feasibility for Real-Time Monitoring of Bacterial Growth in Raw Milk Using a New Contactless Sensor
Source: Anal Chem. 2025 Oct 30;97(44):24438–48. doi: 10.1021/acs.analchem.5c03766 (PMC12613145; doi:10.1021/acs.analchem.5c03766)
Supplement: Supplementary file 1 [file ac5c03766_si_001.pdf]

Supporting Information

Feasibility for Real-Time Monitoring of Bacterial Growth in Raw Milk Using a New Contactless Sensor

Charles A. Haab[a], Jussiane S. Silva[b*], Adriano M. Jaime[a], Vandré S. Pinto[c], Geovana M. Mello[b], Darliana M. Souza[b], Juliano S. Barin[c], Cristiano R. Menezes[c], Leandro Michels[a]

[a] *Departamento de Processamento de Energia Elétrica, Universidade Federal de Santa Maria, 97105-900, Santa Maria, RS, Brazil.*

[b] *Departamento de Química, Universidade Federal de Santa Maria, 97105-900, Santa Maria, RS, Brazil.*

[c] *Departamento de Tecnologia e Ciência dos Alimentos, Universidade Federal de Santa Maria, 97105-900 Santa Maria, RS, Brazil*

*Corresponding author: Tel.+55 55 3220 8802

E-mail address (Jussiane S. Silva): *jussiane.silva@ufsm.br*

Table of contents

Official Methods for Chemical Composition Analysis of Raw Milk (S1.)… S3

Electrical bacterial growth sensor based on a capacitively coupled contactless resonance frequency detector (EBGS-RFD, Auftek, Brazil) used for real-time quantification of total bacterial count (TBC) in raw milk (Figure S1).. S4

Information entered into the AGREE software as input data about the sample preparation methods used (Table S1)... S5

References..S6

S1. Official Methods for Chemical Composition Analysis of Raw Milk

The determination of the chemical composition of the raw milk samples was performed according to the official methods established by the Brazilian Ministry of Agriculture, Livestock, and Supply (MAPA; Normative Instruction No. 77/2018).[1] The specific international standards used for each parameter are detailed below.

Protein Content Determination

The total protein content was determined based on the quantification of total nitrogen according to the Kjeldahl method, as described in ISO 8968-1:2014/IDF 20-1:2014, *Milk and milk products - Determination of nitrogen content, Part 1: Kjeldahl principle and crude protein calculation*. A nitrogen-to-protein conversion factor of 6.38 was used.[2]

Fat Content Determination

The fat content was determined using a gravimetric method following the procedures outlined in ISO 19662:2018/IDF 238:2018, *Milk - Determination of fat content - Acido-butyrometric (Gerber method)*.[3]

Carbohydrate Content Determination

The carbohydrate content was determined by the quantification of lactose, the primary carbohydrate in milk, following the methodology specified in ISO 9622:2013/IDF 141:2013, *Milk and liquid milk products - Guidelines for the application of mid-infrared spectrometry*.[4]

Water Content Determination

The water content was determined indirectly by the gravimetric measurement of total solids, according to ISO 6731:2010/IDF 21:2010, *Milk, cream and evaporated milk - Determination of total solids content (Reference method)*. The sample was dried to a constant weight, and the water content was calculated by difference from the total solids content.[5]

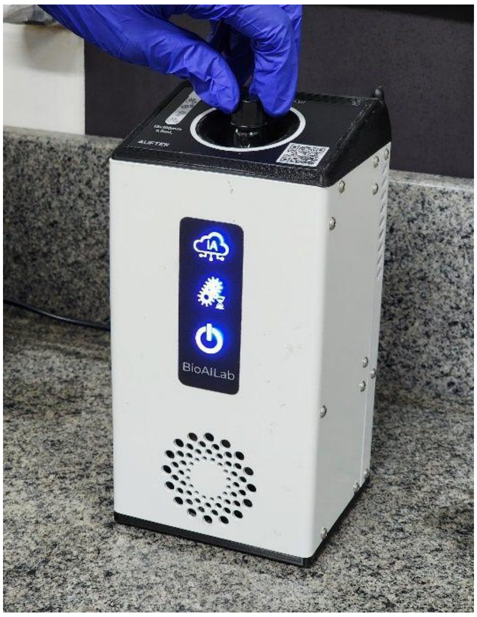

Figure S1. Electrical bacterial growth sensor based on a capacitively coupled contactless resonance frequency detector (EBGS-RFD, Auftek, Brazil) used for real-time quantification of total bacterial count (TBC) in raw milk.

Table S1. Information entered into the AGREE software as input data about the sample preparation methods used.

Principle	Proposed method EBGS-RFD	Reference method SPC
1. Direct analytical techniques should be applied to avoid sample treatment. *Select the sampling procedure:*	At-line analysis	External sample pre-and treatment and batch analysis (large number of steps)
2. Minimal sample size and minimal number of samples are goals. *Enter the amount of sample in either mL:*	7	1
3. If possible, measurements should be performed in situ. *What is the positioning of the analytical device?*	At-line	Off-line
4. Integration of analytical processes and operations saves energy and reduces the use of reagents. *How many major, distinct steps are there in the sample preparation procedure?*	3 or fewer	8 or more
5. Automated and miniaturized methods should be selected. *Degree of automation:/Sample preparation:*	Semi-automatic/Miniaturized	Manual/Not miniaturized
6. Derivatization should be avoided. *Select derivatization agents (if used):*	Not applied	Not applied
7. Generation of a large volume of analytical waste should be avoided, and proper management of analytical waste should be provided. *Enter the amount of waste in mL:*	0	350
8. Multi-analyte or multi-parameter methods are preferred versus methods using one analyte at a time. *Number of analytes determined in a single run: /Sample throughput (samples analysed per hour):*	1 analyte /0.12	1 analyte /0.02
9. The use of energy should be minimized. *Estimate the total power consumption of a single analysis in kWh:*	< 0.1 kWh per sample	> 1.5 kWh per sample
10. Reagents obtained from renewable sources should be preferred. *Select the type of reagents:*	No reagents	All reagents are bio-based
11. Toxic reagents should be eliminated or replaced. *Does the method involve the use of toxic reagents or solvents?*	No	No
12. Operator safety and environmental hazards	Not applied	Not applied

References

(1) MAPA. *Normative Instruction No. 77, November 26, 2018 (in portuguese)*. Brasília, Brazil. https://www.in.gov.br/materia/-/asset_publisher/Kujrw0TZC2Mb/content/id/52750141/do1-2018-11-30-instrucao-normativa-n-77-de-26-de-novembro-de-2018-52749887 (accessed 2025-08-13).

(2) ISO 8968-1:2014/IDF 20-1:2014. *Milk and milk products - Determination of nitrogen content, Part 1: Kjeldahl principle and crude protein calculation*. https://www.iso.org/standard/61020.html (accessed 2021-09-05).

(3) ISO 19662:2018/IDF 238:2018. *Milk - Determination of fat content - Acido-butyrometric (Gerber method)*. https://www.iso.org/standard/65935.html (accessed 2021-09-05).

(4) ISO 9622:2013/IDF 141:2013. *Milk and liquid milk products - Guidelines for the application of mid-infrared spectrometry*. https://www.iso.org/standard/56874.html (accessed 2021-09-05).

(5) ISO 6731:2010/IDF 21:2010. *Milk, cream and evaporated milk - Determination of total solids content (Reference method),*. https://www.iso.org/standard/56815.html (accessed 2023-02-10).
